# Entrainment Ranges for Chains of Forced Neural and Phase Oscillators

**DOI:** 10.1186/s13408-016-0038-9

**Published:** 2016-04-18

**Authors:** Nicole Massarelli, Geoffrey Clapp, Kathleen Hoffman, Tim Kiemel

**Affiliations:** Department of Mathematics and Statistics, University of Maryland, Baltimore County, 1000 Hilltop Circle, Baltimore, MD 21250 USA; Department of Mathematics, University of Maryland, College Park, MD 20742 USA; Department of Kinesiology, University of Maryland, College Park, MD 20742 USA

**Keywords:** Entrainment range, Central pattern generator, Locomotion

## Abstract

Sensory input to the lamprey central pattern generator (CPG) for locomotion is known to have a significant role in modulating lamprey swimming. Lamprey CPGs are known to have the ability to entrain to a bending stimulus, that is, in the presence of a rhythmic signal, the CPG will change its frequency to match the stimulus frequency. Bending experiments in which the lamprey spinal cord has been removed and mechanically bent back and forth at a single point have been used to determine the range of frequencies that can entrain the CPG rhythm. First, we model the lamprey locomotor CPG as a chain of neural oscillators with three classes of neurons and sinusoidal forcing representing edge cell input. We derive a phase model using the connections described in the neural model. This results in a simpler model yet maintains some properties of the neural model. For both the neural model and the derived phase model, entrainment ranges are computed for forcing at different points along the chain while varying both intersegmental coupling strength and the coupling strength between the forcer and chain. Entrainment ranges for chains with nonuniform intersegmental coupling asymmetry are larger when forcing is applied to the middle of the chain than when it is applied to either end, a result that is qualitatively similar to the experimental results. In the limit of weak coupling in the chain, the entrainment results of the neural model approach the entrainment results for the derived phase model. Both biological experiments and the robustness of non-monotonic entrainment ranges as a function of the forcing position across different classes of CPG models with nonuniform asymmetric coupling suggest that a specific property of the intersegmental coupling of the CPG is key to entrainment.

## Introduction

The central pattern generator (CPG) for vertebrate locomotion consists of a circuit of neurons in the spinal cord that produces the basic oscillatory rhythmic output necessary for locomotion such as walking and swimming [1]. Sensory input is known to have a significant effect on the rhythmic output of the CPG in order to adjust to perturbations from the body and environment as well as to adjust the timing of the electrical waves of activity relative to the muscle activity down the body [2–4]. For example, edge cells are stretch receptors located on the margin of the spinal cord of the lamprey that inhibit contralaterally and excite ipsilaterally [3, 5]. Experiments of Tytell and Cohen [6] specifically address the role of edge cells in modulating CPG rhythm (also see [7, 8]). In the presence of a rhythmic stimulus, the vertebrate CPG frequency tends to approach the frequency of that stimulus, a phenomenon known as entrainment. Consider a CPG oscillating at a frequency *ω* in the absence of sensory input. Then consider a CPG subjected to a rhythmic stimulus at a frequency $\omega_{\mathrm{f}}$, close to *ω*. Denote by $\omega^{*}_{i}$ the average frequency of the *i*th oscillator in the chain during forcing, which may or may not be equal to the forcing frequency $\omega_{\mathrm{f}}$. When the CPG’s response is periodic with its frequency equal to the forcing frequency, that is, $\omega_{i}^{*}=\omega_{\mathrm{f}}$ for all *i*, the CPG is said to be *1:1 entrained*. In this paper we only consider 1:1 entrainment, which we will refer to simply as entrainment. The range of frequencies for which the CPG is entrained to the forcer is termed the *entrainment range*. Tytell and Cohen [6] found that the experimental entrainment ranges were approximately twice as large for bending stimuli applied near the middle of the preparation as those for stimuli applied at the ends. This experimental result motivated the study of entrainment in CPG models in order to determine the mechanisms responsible for entrainment ranges which are non-monotonic as function of the forcing position.

The locomotor CPG is commonly represented by a chain of coupled oscillators. Each individual oscillator can be represented by models with varying biological detail (see, for example, [9–13]). The simplest model, a phase model with sinusoidal coupling functions, was pioneered by Cohen, Holmes, and Rand [9] in terms of the lamprey locomotion CPG and represents each oscillator as a single variable. In this paper we refer to this model as the sinusoidal phase model. A neural network model of a segment of the CPG represents classes of neurons in that segment, with the number of variables proportional to the number of classes. Finally, a Hodgkin–Huxley type oscillator models neurons in each segment with multiple physiological variables. Seminal work of Cohen, Holmes, and Rand [9] inspired models of entrainment of forcing at either end of the chain [10, 13, 14]. Previte et al. [15] considered entrainment ranges for chains of phase oscillators, with sinusoidal coupling, forced at any point along the chain. They developed analytical bounds on the entrainment ranges and characterized loss of entrainment.

Here we further investigate the hypothesis from Previte et al. [15] that entrainment ranges that vary non-monotonically as a function of the stimulus position provide information regarding how intersegmental connection strengths vary as a functions of the length and direction. We replace the sinusoidal phase model of [15] with a neural model for each CPG segment and compute entrainment ranges. We further derive a phase model, which incorporates biological details from the neural model into the coupling functions. The resulting phase model (implicitly) contains more biological details than the sinusoidal phase model, but it is simpler than the neural model. We numerically compute entrainment ranges as a function of both stimulus position and forcing strength for the neural and derived phase models. We compare entrainment ranges for the two models, which are predicted to coincide in the limit of weak intersegmental coupling and forcing [9, 16, 17]. Deriving the phase model allows us to determine the extent to which its coupling functions and forcing functions are approximately sinusoidal. If the derived phase model is approximately sinusoidal, this would suggest that the previous analysis of [15] may be sufficient to understand the entrainment of the neural model in the limit of weak coupling. If not, the derived phase model would serve to motivate future analysis that extends the analysis of [15] to a wider range of coupling and forcing functions. Finally, after computing entrainment ranges, we classify how entrainment is lost.

Swimming is a closed-loop system that requires sensory feedback. A powerful approach to study such a closed-loop system is to conduct experiments on its components under open-loop conditions [18]. System identification, parametric and non-parametric modeling, and concepts from control theory can then be used to understand how the open-loop properties of a system’s component determine its closed-loop behavior [18, 19]. This approach has been used to study, for example, blowflies [20] and electric fish [21, 22] and motivates our interest in the open-loop effect of bending on the lamprey CPG.

The manuscript is organized as follows. Section 2 contains a description of the neural model of Buchanan [23] and Williams [24], and it extends the model to include edge cells. Section 3 contains a description of the derivation of the phase model from the more detailed neural model. Entrainment ranges as a function of the connection strength and forcing position are presented in Sect. 4. Loss of entrainment is discussed for both models in Sect. 5. Section 6 contains a comparison of the entrainment results from the sinusoidal phase model, the neural model, and the experimental data.

## Neural Model

**Fig. 1:**
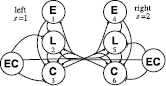
Cell classes of the neural model described in [23, 24] are excitatory interneurons (*E*), lateral inhibitory interneurons (*L*), crossed inhibitory interneurons (*C*), and edge cells (*EC*). *Numbers* indicates cell indices. *Bars* and *circles* indicate excitatory and inhibitory connections, respectively. Edge cells are only active in the segment at which bending occurs

The neural model of the lamprey CPG is based on the model developed by Buchanan [23] and Williams [24]. The model consists of a chain of coupled identical segmental oscillators, with each oscillator corresponding to one anatomical segment of the lamprey spinal cord. The segmental oscillators are modeled as in [24], except that we use a smooth approximation of the piecewise-linear threshold function of [24] (see below). Each segment is described by six variables representing the six classes of cells depicted in Fig. 1. Coupling connections exists between all oscillators, but the strength of the connections depends on their length and direction. Each segment of the CPG consists of three types of neurons: excitatory (E), lateral inhibitory (L), and crossed inhibitory (C) interneurons. Each segment exhibits left–right symmetry with each side containing one E, L, and C cell connected through intrasegmental connections, as illustrated in Fig. 1. Following [12], the effect of bending on the CPG is mediated by edge cells in the margin of the spinal cord, with connections onto CPG cells as shown in Fig. 1 [5]. We model the bending experiments of Tytell and Cohen [6] by assuming that bending activates the edge cells of only one segment.

The model is connectionist with one variable per cell: $v_{ij}$ is the “voltage” of cell *j* in segment *i*, scaled to be unitless and lie between −1 and 1. (For convenience we use the term “cell” to refer to a class of cells.) When $v_{ij} < 0$ the cell does not fire action potentials and $v_{ij}$ represents the membrane voltage of the cell body. When $v_{ij} > 0$ the cell fires action potentials and $v_{ij}$ represents the normalized firing rate. Although the model is connectionist, its form is similar to conductance-based models such as the Hodgkin–Huxley model [25] with the time derivative of voltage proportional to the sum of “currents”, each with its own reversal potential. The reversal potentials are in the range from −1 to 1, so that voltage remains in this same range. The model is

1a $$\begin{aligned} \dot{v}_{ij} = & -G_{R}v_{ij} + G_{T}^{j}(1 - v_{ij}) + \sum _{k=1}^{n} \sum_{l=1}^{6} \alpha_{i-k}^{lj} G_{0}^{lj}h(v_{kl}) \bigl(V_{\mathrm{syn}}^{l} - v_{ij}\bigr) \\ &{}+ \delta_{im} \alpha_{\mathrm{f}} \sum _{s=1}^{2} G_{\mathrm{f}}^{sj} h \bigl(v_{\mathrm{ec}}^{s}(\theta_{\mathrm{f}})\bigr) \bigl(V_{\mathrm{syn},\mathrm {ec}}^{sj} - v_{ij}\bigr), \\ &\hspace{-12pt}\mbox{for }i=1,\ldots,n; j=1,\ldots,6, \end{aligned}$$

1b $$\begin{aligned} \dot{\theta}_{\mathrm{f}} =& \omega_{\mathrm{f}}, \end{aligned}$$

where

1c $$ h(x) = \sigma\log \bigl(1 + e^{{x}/{\sigma}} \bigr) $$

is a smooth threshold function and

1d $$ v_{\mathrm{ec}}^{s}(\theta_{\mathrm{f}}) = (-1)^{s} \sin(2\pi\theta _{\mathrm{f}}) $$

is the edge cell voltage with *s* denoting the left or the right side as illustrated in Fig. 1. (See Table 1 for a list of the model parameters and their values.) In Eqs. (1a)–(1d), *n* represents the number of spinal cord segments in the experimental preparation being modeled. We choose $n = 10$ as a compromise between required computation time and approximating the large number of segments in experimental preparations, where *n* can approach 50. On the right side of (1a), the first term represents the resting conductance that drives the voltage toward 0. The second term represents the tonic excitatory conductance that drives the voltage toward 1. The third term, the double summation, represents the influence of other neurons on $v_{ij}$, which occurs via the intrasegmental ($k = i$) and intersegmental ($k \neq i$) connections. The term $\alpha_{i-k}^{lj} G_{0}^{lj}$ is the maximal synaptic conduction of the connection from cell *l* of oscillator *k* to cell *j* of oscillator *i*. Cell indices are indicated in Fig. 1. Note that the maximal synaptic conductance does not depend on the absolute positions of the two oscillators in the chain, but only on the signed distance between them, $r=i-k$. Note for convenience, we refer to *r* as the connection length, where negative values correspond to ascending connections and positive values correspond to descending connections. For intrasegmental connections, $i=k$, $\alpha_{i-k}^{lj} = 1$ and $G_{0}^{lj}$ is the maximal synaptic conductance. For intersegmental connections, $\alpha_{r}^{lj}$ expresses the maximal synaptic conductance as a fraction of the maximal synaptic conductance of the intrasegmental connection of the same type. Figure 5 illustrates the synaptic conductances for connections between E and C cells, L and C cells, and all other cellular connections. We refer to $\alpha_{r}^{lj}$ as connection strength and describe how connection strengths are specified when we consider the phase-model approximation in Sect. 3. The threshold function *h* given by (1c) describes how coupling depends on the voltage of the presynaptic cell. This function represents an activation threshold, where once the voltage of the neuron reaches a certain threshold it becomes “active.” In contrast to the models of Buchanan [23] and Williams [24], which use a piecewise-linear *h*, we chose a smooth *h* to facilitate our computational analysis. As *σ* decreases to 0, the smooth function approaches the non-smooth version of [23] and [24]. We used $\sigma=0.05$ in our simulations. A connection between cells drives the postsynaptic cell’s voltage toward the synaptic reversal potential $V^{l}_{\mathrm{syn}}$, which depends on the type of the presynaptic cell *l*. If cell *l* is an E cell, which is excitatory, then $V^{l}_{\mathrm{syn}}=1$; if cell *l* is an L or C cell, which are inhibitory, then $V^{l}_{\mathrm{syn}}=-1$.

**Table 1:** **Neural model parameters used for simulations and to compute the derived phase model**

Parameter	Description	Value	Restrictions
*n*	Number of segmental oscillators	10	
*m*	Index of forced oscillator	Varies	1 ≤ *m* ≤ *n*
$G_{R}$	Resting conductance	3.5 s^−1^	
$G^{j}_{T}$	Tonic excitatory conductance	0.875 s^−1^	E cells
		0.350 s^−1^	L cells
		3.500 s^−1^	C cells
$G^{kl}_{0}$	Maximal synaptic conductance of intersegmental connection	15 s^−1^	L to C connection
		35 s^−1^	All other connections
$V^{l}_{\mathrm{syn}}$	Synaptic reversal potential for intersegmental connection	1	Excitatory connections
		−1	Inhibitory connections
*σ*	Smoothing parameter of threshold function	0.05	
$\alpha_{r}^{lj}$	Intersegmental connection strength	See Fig. 5	
$A_{d}$	Amplitude of descending coupling	Varies	
$A_{a}$	Amplitude of ascending coupling	Varies	
$\lambda_{d}$	Length constant of descending coupling	Varies	
$\lambda_{a}$	Length constant of ascending coupling	Varies	
$\alpha_{\mathrm{f}}$	Forcing strength	Varies	
$\omega_{\mathrm{f}}$	Forcing frequency	Varies	
$V^{sj}_{\mathrm{syn},\mathrm{ec}}$	Synaptic reversal potential for EC connection	1	Excitatory connections
		−1	Inhibitory connections
$G^{sj}_{\mathrm{f}}$	Maximal synaptic conductance of EC connections	1	

The last term of (1a), the single summation, describes the influence of bending via edge cells on the CPG voltages $v_{ij}$. We use the Kronecker delta function $\delta_{im}$ to indicate that bending only occurs at segment *m*. The summation index *s* indicates whether input is from the edge cell on the left ($s = 1$) or right ($s = 2$) side. The parameter $\alpha_{\mathrm{f}}$ is the strength of forcing and the parameters $G_{\mathrm{f}}^{sj}$ are used to indicate the relative strength of forcing on different cells in segment *m*. For simplicity, we assume that $G_{\mathrm{f}}^{sj} = 1$ for all the edge cell connections shown in Fig. 1 and $G_{\mathrm{f}}^{sj}=0$ otherwise. The parameter $V_{\mathrm{syn} ,\mathrm{ec}}^{sj}$ is the synaptic reversal potential for the connection from the edge cell on side *s* to cell *j*; $V_{\mathrm{syn},\mathrm{ec}}^{sj}$ is 1 for the ipsilateral connections, which are excitatory, and −1 for the contralateral connections, which are inhibitory. For an edge cell connection, the input to the threshold function *h* is the voltage $v_{\mathrm{ec}}^{s}(\theta_{\mathrm{f}})$, which is defined by (1b) and (1d).

## Derived Phase Model

To test how coupling asymmetry affects the shape of entrainment ranges as a function of the forcing position we study another phase model which is derived from the neural model described in Sect. 2. A phase model is a simplification of the neural model and represents each anatomical segment of the CPG with a single variable. Previte et al. [15] studied a phase model with sinusoidal coupling functions. However, instead of using sine functions to couple the oscillators, we use the neuron-to-neuron connections in the neural model to compute intersegmental connections between oscillators. We exploit the theory of weakly coupled oscillators [9, 16, 17] to approximate the neural model given by (1a)–(1d) by a phase model of the form

2a $$\begin{aligned} \dot{\theta}_{i} = &\omega_{0} + \sum _{\substack{k=1\\k \neq i}}^{n} \sum_{j=1}^{6} \sum_{l=1}^{6} \alpha_{i-k}^{lj} H^{lj}(\theta_{k} - \theta_{i}) \\ &{}+ \delta_{im} \alpha_{\mathrm{f}} \sum_{s=1}^{2} \sum_{j=1}^{6} H_{\mathrm{f}}^{sj}( \theta_{\mathrm{f}} - \theta_{i}), \quad\mbox{for } i=1,\ldots,n, \end{aligned}$$

2b $$\begin{aligned} \dot{\theta}_{\mathrm{f}} = &\omega_{\mathrm{f}}, \end{aligned}$$

under the assumptions that intersegmental connection strengths $\alpha_{r}^{lj}$ ($r \neq0$) and forcing strength $\alpha _{\mathrm{f}}$ are small and $\omega_{\mathrm{f}}$ is close to *ω*. The function $H^{lj}$ describes the coupling provided by a single intersegmental connection of unit strength from cell *l* in one segment to cell *j* in another segment. Similarly, $H_{\mathrm{f}}^{sj}$ describes the coupling provided by a connection of unit strength from the edge cell on side *s* of segment *m* to cell *j* in the same segment. Note we no longer consider intrasegmental coupling since each segment is represented by a single variable.

**Fig. 2:**
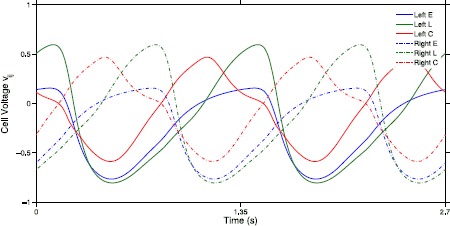
Simulation of a single segment within a chain of oscillators defined by (1a)–(1d) for two cycles without forcing. Plot shows the cell voltages within the first segment ($i=1$). Weak intersegmental coupling, defined by $A_{a}=0.0004$, $A_{d}=0.0002$, $\lambda_{a}=\lambda_{d}=4$, was used to connect segments. Thus, the solution for the oscillator in the chain closely approximates the solution for a single, uncoupled oscillator. Note the spatiotemporal symmetry between left and right cells. The voltage of the left cells is the same as the voltage of the right cells except for a phase shift of half a period

Recall that intersegmental connections have the same connectivity as the intrasegmental connections shown in Fig. 1. For example, given coupling length *r*, there are 12 nonzero $\alpha_{r}^{lj}$ corresponding to 2 connections for each of 6 connection types: E to C, E to L, L to C, C to E, C to L, and C to C. Due to the right–left symmetry of the neural model and the left–right spatiotemporal symmetry of the segmental oscillator’s limit cycle, two connections of the same type have the same connection strength and same coupling function. Note these symmetries can be seen in Fig. 2, which depicts the steady state of the neural model for one segment, simulated without forcing. The left and right cells have the same voltage with a phase shift of half a period. Therefore, we can write

3 $$ \sum_{j=1}^{6} \sum _{l=1}^{6} \alpha_{r}^{lj} H^{lj} = \sum_{c \in\mathcal{C}} \alpha_{r,c} H_{c}, \quad \mbox{where $\mathcal{C} = \{\mathrm{EL},\mathrm{EC},\mathrm{LC},\mathrm{CE},\mathrm{CL},\mathrm{CC}\}$} $$

where, for example, $\alpha_{r,\mathrm{EL}} = \alpha_{r}^{12} = \alpha_{r}^{45}$ and $H_{\mathrm{EL}} = H^{12} + H^{45} = 2H^{12}$. Let $\alpha_{r}$ be the mean of $\alpha_{rc}$ for $c \in\mathcal{C}$. We define $H_{r}$, the coupling function of the length *r*, as

4 $$ H_{r} = \frac{1}{\alpha_{r}} \sum_{j=1}^{6} \sum_{l=1}^{6} \alpha_{r}^{lj} H^{lj} = \sum_{c \in\mathcal{C}} \frac{\alpha_{r,c}}{\alpha_{r}} H_{c}. $$

Similarly, the 8 edge cell connections of Fig. 1 consist of two connections for each of the four connection types: EC to Li, EC to Ci, EC to Lc, and EC to Cc, where ‘i’ and ‘c’ indicate ipsilateral and contralateral connections, respectively. Therefore, we can define the forcing coupling function $H_{\mathrm{f}}$ as

5 $$ H_{\mathrm{f}} = \sum_{s=1}^{2} \sum _{j=1}^{6} H_{\mathrm{f}}^{sj} = H_{\mathrm{f},\mathrm{Li}} + H_{\mathrm{f},\mathrm{Ci}} + H_{\mathrm{f},\mathrm{Lc}} + H_{\mathrm{f},\mathrm{Cc}}, $$

where, for example, $H_{\mathrm{f},\mathrm{Li}} = H_{\mathrm{f}}^{11} + H_{\mathrm{f}}^{25} = 2 H_{\mathrm{f}}^{11}$.

Now, using (4) and (5), we can write the phase model (2a), (2b) as

6a $$\begin{aligned} \dot{\theta}_{i} &= \omega_{0} + \sum _{\substack{k=1\\k \neq i}}^{n} \alpha_{i-k} H_{i-k}( \theta_{k} - \theta_{i}) + \delta_{im} \alpha_{\mathrm{f}} H_{\mathrm{f}} (\theta_{\mathrm {f}} - \theta_{i}), \quad\mbox{for }i=1,\ldots,n, \end{aligned}$$

6b $$\begin{aligned} \dot{\theta}_{\mathrm{f}} &= \omega_{\mathrm{f}}. \end{aligned}$$

Model (6a), (6b) has the standard form of a chain of coupled phase oscillators forced at one location. To specify this model, two choices remain. First, for each connection length *r* we must specify the connection strength ratios $\alpha_{r,c}/\alpha_{r}$ in (4) that determine the coupling function $H_{r}$. We defer this specification until we have computed the coupling function $H_{c}$ for each connection type *c* (see Fig. 4 below). Second, we must specify how coupling strength $\alpha_{r}$ depends on *r*. Experimental evidence does not provide the exact form of this dependence but does indicate an asymmetry in ascending and descending coupling strengths [7, 26, 27]. Among the possible modeling choices in the literature (e.g. [11, 28]), we will follow Varkonyi et al. [29] and assume that the coupling strength decays exponentially with coupling length:

7 $$ {\alpha}_{r} = \textstyle\begin{cases} A_{d} e^{-|r|/\lambda_{d}} & \mbox{for $r>0$ (descending connections),} \\ A_{a} e^{-|r|/\lambda_{a}} & \mbox{for $r< 0$ (ascending connections),} \\ 1 & \mbox{for $r=0$ (intrasegmental connections),} \end{cases} $$

where $A_{d}$, $\lambda_{d}$ and $A_{a}$, $\lambda_{a}$ are the amplitudes and length constants for descending and ascending coupling, respectively. Representative parameter values can be found in the caption of Fig. 8.

### Coupling Functions

To define the functions $H_{r}$ and $H_{\mathrm{f}}$ in (6a), (6b) we use the methods of phase reduction and averaging (see [30–32]) as applied to weakly coupled oscillators [29]. Under the assumption of weak coupling in (1a)–(1d), we can describe the intrasegmental connections in the neural model as a phase dependent coupling function for each connection type, $H_{c}$.

**Fig. 3:**
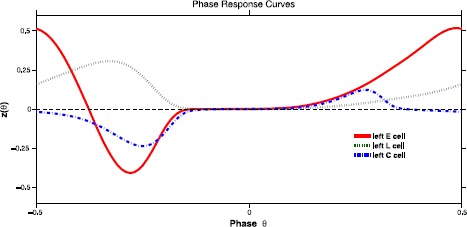
The PRCs are plotted for the left E, L, and C cells. Each PRC describes the resulting phase shift that occurs when that cell’s voltage is perturbed by 10^−6^, at various initial phases. PRCs for right E, L, and C cells are the same except for a phase shift of 0.5 due to the right–left symmetry within each oscillator

**Fig. 4:**
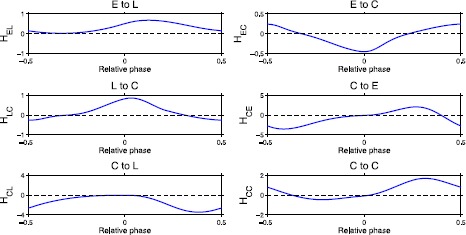
For each type of neural connection between E, L, and C cells, an $H_{c}$ function is computed to represent the effects of neurons on the voltage of the neuron within the oscillator. The six $H_{c}$ functions are computed for connections from L to E cells, C to E cells, C to L cells, E to C cells, L to C cells, and C to C cells. Here we show only half of the neuron-to-neuron connections in Fig. 1 because of the left–right symmetry within the oscillator

Applying the techniques used in [29] to (1a)–(1d) the 6 intersegmental coupling functions $H_{c}$ in (3) are computed. The first step in this process is to compute the phase response curves (PRCs) for each class of neurons within a single segment. Figure 3 illustrates the PRCs for the neural model (1a)–(1d). These are the 6 neuron-to-neuron connections in half of a single oscillator. Recall that due to the spatiotemporal symmetry (seen in the connections in Fig. 1 and the simulated voltages in Fig. 2) $H_{c}$ are the same for connections between neurons on the right side of the oscillator and those on the left side. The six connections for the left E, L, and C cells are depicted in Fig. 4.

**Fig. 5:**
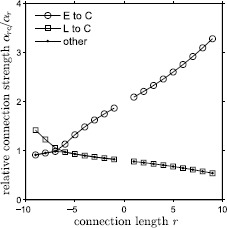
Relative strengths $\alpha_{rc}/\alpha_{r}$ of different connection types as a function of the connection length *r*

Recall that the intersegmental coupling functions defined by (4) are a linear combination of the six neuron-to-neuron connections $H_{c}$. In (4), $\alpha_{rc}$ determines how much each neuron-to-neuron connection of length *r* contributes to the intersegmental connection for oscillators *i* and *k* where $r=i-k$. The choice of $\alpha_{rc}$ also determines the phase lag between oscillators. Experimentally, a phase lag of approximately 1% of the cycle per segment has been observed [1, 33]. This means that as neural activity travels down the CPG, the phase difference between consecutive segments is 0.01. Choosing the correct set of coefficients to produce the desired phase lag is called tuning. We use the tuning methods in [26] to determine the appropriate $\{\alpha_{rc}\}$. Tuning is achieved when the zeros of the coupling functions match the phase lag of 0.01 per segment. After tuning, for a chain of ten oscillators, we have 18 intersegmental connection functions $H_{r}$ for $r=-9,\ldots,-1,1,\ldots,9$ representing both ascending and descending connections. Each $H_{r}$ is then multiplied by ${\alpha}_{r}$, the average of the intrasegmental connection strengths of length *r*. The fraction of the connection strength $\alpha_{rc}/\alpha_{r}$ is depicted in Fig. 5 for the different cell-to-cell connections.

**Fig. 6:**
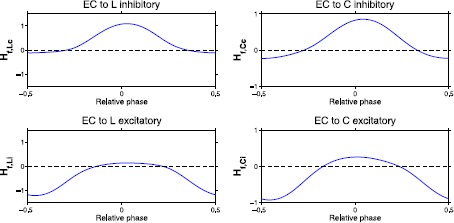
For each type of neural connection from edge cells, an $H_{\mathrm{f},c}$ function is computed that describes the strength of that connection as a function of the relative phase between the edge cell and the oscillator where forcing is applied

A method similar to the one used to compute intersegmental coupling functions $H_{r}$ is used to compute $H_{\mathrm{f}}$, where cell *i* is replaced by an edge cell. Hence, $\theta_{i}$ will represent the phase of the forcer, which has its own period $T_{\mathrm{f}}=1/ \omega_{\mathrm{f}}$. These edge cell connections are depicted in Fig. 6 for the left edge cell.

As described before by (5), the forcing function $H_{\mathrm{f}}$ in (6a), (6b) is defined as the sum of all of the edge cell connections. Here we assume that each edge cell connection contributes equally to the overall forcing connection (each function has coefficient 1).

At this point, we have computed all components of the phase model: intersegmental connections, $H_{r}$, and forcing connection, $H_{\mathrm{f}}$. However, rather than use the phase model directly, we instead consider the relative phase model by looking at the phase difference between each oscillator and the phase of the forcer. This is characterized by the change of variable $\phi_{i} = \theta_{\mathrm{f}}-\theta_{i}$, which transforms (6a), (6b) to

8 $$\begin{aligned} \dot{\phi}_{i} &= \delta - \sum _{\substack{k=1\\k \neq i}}^{n} \alpha_{i-k} H_{i-k}( \phi_{i} - \phi_{k}) - \delta_{im} \alpha_{\mathrm{f}} H_{\mathrm{f}} (\phi_{i}),\quad\mbox{for } i=1,\ldots,n, \end{aligned}$$

where $\delta= \omega_{\mathrm{f}} - \omega_{0}$. In the phase model, entrainment corresponds to stable periodic orbits, whereas in the relative phase model, entrainment corresponds to stable fixed points of (8). When the CPG is entrained to the forcing frequency, the phase difference between a given oscillator in the chain and the forcing oscillator remains constant. Using the relative phase model allows us to use continuation and fixed point stability analysis, which we can exploit to find entrainment ranges.

## Entrainment Ranges

In this section, entrainment ranges are computed as functions of forcing position, forcing strength and intersegmental coupling strength. For the neural model (1a)–(1d), a periodic solution entrained to a given forcing frequency would correspond to a fixed point of the Poincaré map. For the relative phase model, the CPG is entrained when the relative phases, that is, the differences in phase between an oscillator in the chain and the forcing oscillator, $\theta_{\mathrm{f}} - \theta_{i}$, are constant. This implies that all of the oscillators in the chain have the same frequency as the forcer, namely $\omega_{\mathrm{f}}$. Constant relative phases correspond to stable fixed points of (8). For both models, entrainment ranges can be computed by identifying stable fixed points.

Standard parameter continuation methods (see, for example, [34]) are used to track fixed points in dynamical systems in order to determine the boundaries of entrainment ranges. In the simplest case (shown in Sect. 4.2), the parameter $\delta= \omega_{\mathrm{f}} - \omega$ is varied and the lower and upper boundaries of the entrainment range are values of *δ* where the fixed point loses stability. Stability is assessed by computing the eigenvalues of the Jacobian evaluated at the fixed point. In order to determine how the entrainment range varies with forcing strength $\alpha_{\mathrm{f}}$ (shown in Sect. 4.1), we performed a series of one-parameter continuations in order to compute curves in $(\alpha_{\mathrm{f}}, \delta)$ parameter space that correspond to loss of stability.

**Fig. 7:**
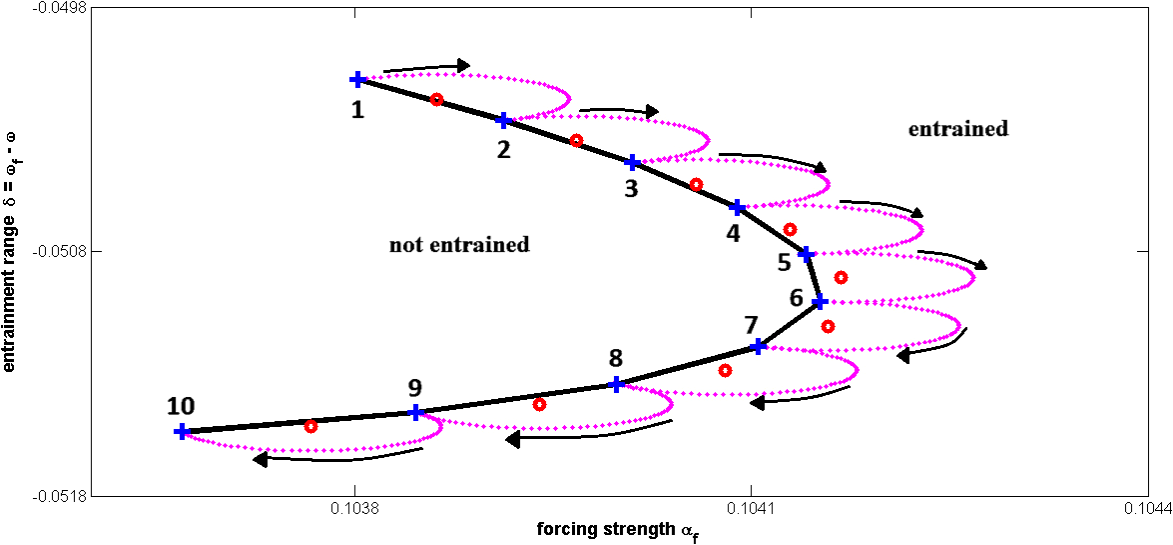
Illustration of two-parameter continuation used to find entrainment ranges as a function of the forcing strength. *The dotted circles* denote how the values of $\delta=\omega_{\mathrm{f}}-\omega_{0}$ and $\alpha_{\mathrm{f}}$ are updated at each continuation step. *The plus signs* denote points on the entrainment range that are detected by the continuation circles. This allows us to detect sharp corners that may be missed with standard continuation where we only look at vertical slices of parameter space

We used a series of one-parameter continuations instead of two-parameter continuations, because a two-parameter continuation can become inaccurate near degenerate bifurcations [35]. In order to follow these curves in any direction in parameter space, the one-parameter continuations were performed along ellipses in parameter space rather than straight lines, as illustrated in Fig. 7. The larger dotted ellipses represent the path of the continuation steps in the parameter space. These ellipses indicate how the parameters $\delta= \omega_{\mathrm{f}} - \omega$ and $\alpha_{\mathrm{f}}$ are updated at each continuation step. We choose the size of the ellipse so that it is large enough to cover a relatively large area in parameter space in order to decrease computation time and also small enough to capture sharp corners of the entrainment range. The small red circles indicate the center of continuation ellipses. To choose the next center, we take a step in the same direction as the previous entrainment point. The points on the entrainment range are indicated by blue plus signs. To better explain this process, consider entrainment points 2 and 3 in Fig. 7. We start with entrainment point 2, which is a known point on the entrainment rage. To get the next center, indicated by the small red circle between points 2 and 3, we step in the same direction as the vector from point 1 to point 2. We then move around the large ellipse, plotted in magenta, and find new fixed points with slightly different values of $\alpha_{\mathrm{f}}$ and *δ*. To determine points on the boundary of the entrainment range, we compute the stability of the fixed points in each model.

### Entrainment Ranges as a Function of Forcing Strength

**Fig. 8:**
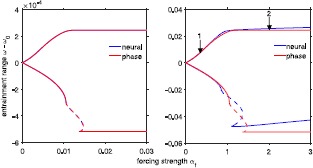
Entrainment ranges for the neural and derived phase models as a function of the forcing strength. *Left* *panel* illustrates entrainment ranges as a function of the forcing strength for weak intersegmental coupling corresponding to $A_{a}=0.0004$, $A_{d}=0.0002$, and $\lambda_{a}=\lambda_{d}=4$. *Right* *panel* illustrates entrainment ranges for 100 times stronger intersegmental coupling with $A_{a}=0.04$ and $A_{d}=0.02$. Note that for weak coupling the entrainment ranges for the neural and derived phase models match closely while for strong coupling the entrainment ranges start to differ as the forcing strength increases. *The* *dashed* *line* on both plots represents Hopf bifurcations that occur when entrainment is lost. *Smooth* *lines* denote saddle-node bifurcations. *The* *arrows* in right panel correspond to the forcing strength values $\alpha _{\mathrm{f}}$ where loss of entrainment is depicted in Figs. 10 and 11

Entrainment ranges are computed for both the neural model and the derived phase model as a function of the forcing strength using our continuation algorithm. Figure 8 illustrates 1:1 entrainment ranges for a chain of ten oscillators forced at the last oscillator as a function of the forcing strength $\alpha_{\mathrm{f}}$. The entrainment range, as a function of the forcing strength, is plotted relative to the unforced, average frequency of the chain that is, the vertical axis represents the difference between the forcing frequency, $\omega_{\mathrm{f}}$ and the natural chain frequency *ω*. Figure 8(left), illustrates entrainment ranges for both the neural model (indicated by the blue line) and the derived phase model (indicated by the red line) for weak intersegmental coupling strength corresponding to $A_{d} = 0.0004$, $A_{a} = 0.0002$, and $\lambda_{d} = \lambda_{a} = 4$ in Eq. (7). Figure 8(right) illustrates entrainment ranges with intersegmental coupling strength 100 times stronger than in Fig. 8(left) ($A_{d} = 0.04$, $A_{a} = 0.02$). Together Figs. 8(left) and 8(right) illustrate the approximate scaling of entrainment ranges with intersegmental coupling strength. For stronger coupling, the derived phase model captures the general properties of the neural entrainment range but not the details as seen in Fig. 8(right). In the limit of weak coupling, as in Fig. 8(left), both the neural model and the derived phase model agree almost exactly, including the type of bifurcation that occurs when entrainment is lost. The smooth lines correspond to saddle-node bifurcations, and the dashed lines represent Hopf bifurcations. For strong coupling, the phase model is not as good of a quantitative approximation of the neural model but does capture the same qualitative features of the entrainment ranges of the neural model, including bifurcation type.

### Entrainment Ranges as a Function of Forcing Position

**Fig. 9:**
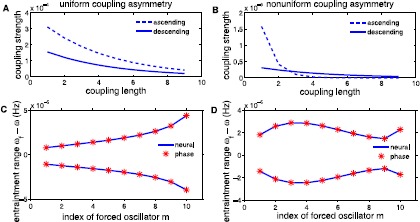
Entrainment ranges as a function of the forcing position for varying intersegmental connections. Uniform coupling asymmetry is illustrated in **A** with $A_{a}=0.0004$, $A_{d}=0.0002$, and $\lambda_{a}=\lambda_{d}=4$. All of the ascending coupling strengths are stronger than descending for all connection lengths. This coupling scheme is used to produce monotonic entrainment ranges as a function of the forcing position, seen in **C**. Nonuniform coupling asymmetry is depicted in **B** with $A_{a}=0.006$, $A_{d}=0.0004$, $\lambda_{a}=0.75$, and $\lambda_{d}=4$. For our choice of parameters, ascending connections become stronger at connections of length 3. Nonuniform coupling is used to compute the entrainment range in **D**, where see non-monotonic entrainment ranges

Figure 9 illustrates the effect of different types of intersegmental coupling on entrainment ranges plotted as a function of the forcing position. Figure 9A shows the strength of the connections plotted as a function of the connection length for both ascending and descending coupling and corresponds to Eq. (7) with parameters $A_{a}=0.0004$, $A_{d}=0.0002$, and $\lambda_{a}=\lambda _{d}=4$. Strength of the ascending connections are uniformly stronger than descending connection strengths, hence we refer to this intersegmental coupling scheme as uniform coupling asymmetry. Similarly, Fig. 9B shows connection strengths, again as a function of the connection length, for both ascending and descending connections where $A_{a}=0.006$, $A_{d}=0.0004$, $\lambda_{a}=0.75$, and $\lambda_{d}=4$. Note in this case, for connections of length 1 and 2, ascending strengths are stronger than descending strengths, but the curves cross transversely (at approximately coupling length 3), after which descending connections become stronger than ascending connections. We refer to this coupling scheme as nonuniform coupling asymmetry.

We consider entrainment ranges as a function of the forcing position to test the hypothesis that nonuniform coupling asymmetry produces larger entrainment ranges when forcing the middle of the chain of oscillators than when forcing at either end. We compute entrainment ranges as a function of the forcing position *m* for the examples of uniform and nonuniform asymmetric coupling illustrated in Fig. 9. Figure 9C and 9D depict entrainment ranges for both the neural (blue line) and the derived phase (stars) models. Note that at the boundary of the entrainment ranges, entrainment is lost externally which means the chain of oscillators has a different average frequency than the forcing oscillator (for more details please see Sect. 5). When the chain has uniform intersegmental coupling asymmetry (Fig. 9A), entrainment range is a monotonically increasing function of the forcing position, as seen in Fig. 9C for both the neural and the derived phase model. When the chain has nonuniform intersegmental coupling asymmetry (Fig. 9B), entrainment range is a non-monotonic function of the forcing position, since the largest entrainment range occurs at $m = 3$, as seen in Fig. 9D. Nonuniform coupling asymmetry produces qualitatively the same entrainment ranges as a function of the forcing position as the experimental data and supports the hypothesis of Previte et al. [15] that non-monotonic entrainment ranges as a function of the forcing position are not a generic property of coupled oscillators but rather depends on intersegmental coupling properties. Further, note that since coupling strength is relatively weak, the phase model acts as a very good approximation of the neural model.

## Loss of Entrainment

To compare with the analytic loss of entrainment results described in [15], we characterize how entrainment is lost outside of the entrainment ranges for the neural and derived phase model. In the sinusoidal phase model, entrainment is lost solely through saddle-node bifurcations. However, in both the neural and the derived phase models entrainment is lost either via a saddle-node bifurcation or a Hopf bifurcation (also known as a Neimark–Sacker bifurcation) of the Poincaré map [30]. Lines of saddle-node and Hopf bifurcations meet at a codimension-two Bogdanov–Takens bifurcation of the Poincaré map [36]. The type of bifurcation varies along the lower branches of the entrainment ranges in Fig. 8. Unlike the entrainment ranges of the sinusoidal phase model of Previte et al. [15], the entrainment ranges of the derived phase model capture the types of bifurcations seen in the entrainment ranges of the neural model.

**Fig. 10:**
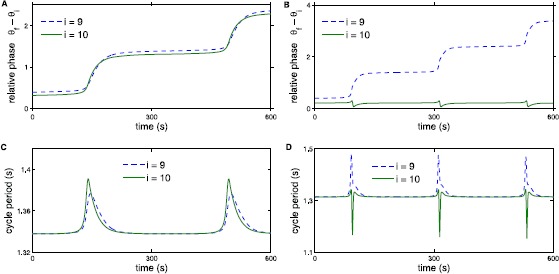
Example of loss of entrainment for the neural model. Panel **A** shows phase relative to the forcer for external loss of entrainment with $\alpha_{\mathrm{f}}=0.5$ and $\omega_{\mathrm{f}}-\omega$ is +0.0002 above the entrainment range as indicated by arrow 1 in Fig. 8(right). Panel **B** shows relative phase for internal loss of entrainment with $\alpha_{\mathrm{f}}=2$ and $\omega _{\mathrm{f}}-\omega$ is +0.0002 above the entrainment range indicated by arrow 2 in Fig. 8(right). Panels **C** and **D** show cycle period for external and internal loss of entrainment, respectively

**Fig. 11:**
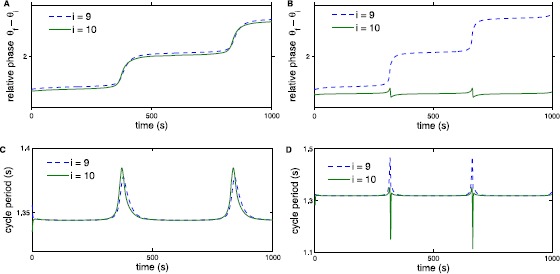
Example of loss of entrainment for the derived phase model. The coupling parameters are the same as those shown in Fig. 10. We see that entrainment is lost in the same way for both the neural and the derived phase model, further supporting that the phase model contains the same entrainment information

Following the definitions of loss of entrainment in [15], we investigate internal versus external loss of entrainment in the CPG models. Internal loss of entrainment occurs when part of the chain follows $\omega_{i}^{*}=\omega_{\mathrm{f}}$ but for the rest of the chain $\omega _{i}^{*}\neq\omega_{\mathrm{f}}$. This split can occur above or below the oscillator where forcing is applied, corresponding to rostral or caudal internal loss of entrainment. External loss of entrainment occurs when $\omega _{i}^{*}$ are equal for all oscillators in the chain but are not equal to the forcing frequency $\omega_{\mathrm{f}}$. Figures 10 and 11 illustrate loss of entrainment for the neural model (Fig. 10) and the derived phase model (Fig. 11) for two values of forcing strength as indicated by the arrows in Fig. 8(right). For small values of the forcing strength $\alpha_{\mathrm{f}}$, the size of the entrainment range increases approximately linearly with $\alpha_{\mathrm{f}}$ as illustrated in Fig. 8 and entrainment at both the lower and the upper limits of the entrainment range is lost via saddle-node bifurcations. For forcing strength sufficiently large, the entrainment range is approximately constant as seen in Fig. 8.

Figure 10A corresponds to simulating the model described by (1a)–(1d) with $\omega_{\mathrm{f}}$ chosen so that $\omega_{\mathrm{f}} -\omega$ is just above (+0.0002) the entrainment range illustrated in Fig. 8(right) for $\alpha_{\mathrm{f}}=0.5$. Figure 10A illustrates that segmental oscillators 9 and 10 are losing one cycle with the forcer. Figure 10C shows a corresponding spike in the cycle period at each step in the relative phase. Simulating with $\alpha_{\mathrm{f}}=0.5$ just below the entrainment range would produce a similar result to Fig. 10A, except the segmental oscillator will gain one cycle with the forcer. The loss of entrainment illustrated in Fig. 10A and 10C corresponds to external loss of entrainment because segments nine and ten (representative of the entire chain) are oscillating together and losing a cycle with the forcer at each step.

Figure 10B also demonstrates loss of entrainment but in this case $\alpha_{\mathrm{f}}=2$ and $\omega_{\mathrm{f}}$ chosen so that $\omega_{\mathrm{f}} -\omega$ is just above (+0.0002) the entrainment range illustrated in Fig. 8(right). Forcing is still on the tenth oscillator, but instead of both oscillators nine and ten losing or gaining a cycle with the forcer at the same time, Fig. 10B shows that oscillator nine is losing a cycle with the forcer, whereas oscillator ten is still oscillating with the forcer. Figure 10D shows a spike in the cycle period as was seen in Fig. 10C at each step in relative phase. This loss of entrainment corresponds to internal loss of entrainment because part of the chain is oscillating at the same frequency as the forcer and another part is not. Internal loss of entrainment can be characterized further as rostral or caudal. Rostral loss of entrainment means that segmental oscillators above the forced oscillator have a different average frequency than the forcer, but the oscillators below the forced oscillator have the same average frequency as the forcer. On the other hand, caudal loss of entrainment means that the loss of entrainment takes place for oscillators below the forced oscillator. Since we consider the case where forcing is applied to the last oscillator in the chain, we can only see rostral loss of entrainment where oscillators 1 through 9 have a different frequency $\omega^{*}_{i}$. The neural model described by (1a)–(1d) exhibits both external loss of entrainment for the entrainment ranges that grow linearly as a function of $\alpha_{\mathrm{f}}$, and internal loss of entrainment where the entrainment ranges are a relatively constant function of $\alpha_{\mathrm{f}}$ (see Fig. 8). The loss of entrainment near the Hopf bifurcation in Fig. 8 is more complex and does not clearly fall into either of these two categories.

Both internal and external loss of entrainment are also seen in the derived phase model. In Fig. 11A, entrainment is lost externally for forcing frequency above the entrainment range for $\alpha _{\mathrm{f}}=0.5$. Figure 11B shows internal loss of entrainment for $\alpha_{\mathrm{f}}=2$. As in the neural model, Figs. 11A and 11B illustrate how the oscillators gain a cycle with the forcer. In Fig. 11A, all 10 oscillators have the same frequency $\omega ^{*}_{i} \neq\omega_{\mathrm{f}}$ while in Fig. 11B, $\omega _{10}^{*}=\omega _{\mathrm{f}}$ but oscillators 1 through 9 have a different frequency. Figures 11C and 11D illustrate the jump in cycle period where the relative phases gain a cycle in relation to the forcing frequency.

In summary, for the upper bound on the entrainment range, entrainment is lost externally for small values of $\alpha_{\mathrm{f}}$ when the entrainment range is growing linearly as a function of $\alpha_{\mathrm{f}}$, whereas entrainment is lost internally in the range of $\alpha_{\mathrm{f}}$ where the entrainment range is relatively constant as a function of $\alpha_{\mathrm{f}}$. In both these ranges, entrainment is lost through a saddle-node bifurcation of the return map in the Poincaré section. Hence, the type of loss of entrainment does not necessarily correspond to the type of bifurcation. Loss of entrainment just below the entrainment range exhibits more complicated behavior which, for some $\alpha_{\mathrm{f}}$ values, cannot easily be classified as internal or external. Finally, the derived phase model agrees with the neural model on how entrainment is lost at different locations along the entrainment range. This further illustrates that the derived phase model preserves entrainment information as regards the more biologically detailed neural model.

## Discussion

The lamprey central pattern generator for locomotion is considered to be a model system for studying vertebrate locomotion because it is a primitive vertebrate with relatively few neurons [37, 38]. Another advantage of studying the lamprey central pattern generator for locomotion is that the spinal cord of the lamprey can be excised from the animal, placed in a bath of the excitatory amino-acid d-glutamate and still produce motor nerve activity similar to that of a swimming lamprey. Tytell and Cohen [6] measured entrainment ranges for bending at different locations along a roughly 50-segment piece of spinal cord and found that entrainment ranges were larger in middle of the piece than at either end.

The dependence of the effect of bending on location along the spinal cord could be due to some combination of the properties of intersegmental coupling, as modeled by Previte et al. [15], or differences in the local effect of bending, as suggested by Hsu et al. [39]. Motivated by the work of Previte et al., we investigated the effect of intersegmental coupling on entrainment properties of both a neural model and its phase-model approximation. As expected based on the theory of phase reduction for weakly coupled oscillators, we saw the entrainment characteristics of the neural model were closely approximated by the derived phase model in the limit of weak coupling. This included entrainment ranges as a function of the forcing strength, entrainment ranges as a function of the position, and also loss of entrainment. Additionally, we computed entrainment ranges as a function of the forcing position with different coupling schemes. For both the neural and the derived phase model we saw monotonic and non-monotonic entrainment ranges as a function of the forcing position for uniform and nonuniform coupling asymmetry, respectively. Entrainment is also lost in the same way in both models as illustrated by Figs. 10 and 11. Comparing the entrainment results for the neural and derived phase models indicates that the derived phase model is able to capture all of the essential entrainment properties we analyzed. Thus, with sufficiently weak coupling, entrainment can be studied in the simpler derived phase model.

Previous analytic results only considered internal loss of entrainment in a phase model [13]. Previte et al. [15] characterized loss of entrainment for the sinusoidal phase model, as either internal or external as described in Sect. 5. Previte et al. [15] also showed that internal loss of entrainment is more likely when forcing strength $\alpha_{\mathrm{f}}$ is strong relative to coupling strengths $\alpha_{r}$. Our simulations in Figs. 10 and 11 support this conclusion. For relatively weak forcing strength, $\alpha_{\mathrm{f}}=0.5$, entrainment is lost externally for both the neural and the derived phase models. Alternatively, for stronger forcing strength, $\alpha_{\mathrm{f}}=2$, entrainment is lost internally where oscillator 9 has a different frequency than oscillator 10. These results support the claim that experimental entrainment needs to be re-examined to determine how entrainment is lost at the middle and ends of the chain [15]. Experimental procedures make it difficult to classify exactly how entrainment is lost. Moreover, experimental entrainment ranges plot the average frequency of the oscillators in the chain, which obscures more subtle differences [15].

Although both chains of coupled oscillators, the neural and derived phase models contain different levels of biological detail in comparison to the simpler sinusoidal phase model. Despite these differences, entrainment results are qualitatively similar across all three models. Entrainment ranges as a function of the forcing position are plotted in Fig. 9 for both the neural and the derived phase models. We see similarly shaped entrainment ranges as a function of the forcing position in our two models as well as the sinusoidal phase model studied by Previte et al. [15]. This supports the hypothesis that non-monotonic entrainment ranges are not an intrinsic property of chains of coupled oscillators but rather a characteristic of a specific type of intersegmental coupling. Specifically, nonuniform coupling asymmetry, in each model, produces entrainment ranges that do not increase monotonically as forcing position increases. Additionally, computational and experimental results have indicated coupling asymmetry exists in the lamprey CPG, but the strength and direction of the connections is still unknown [26, 40]. More recently, experiments have been conducted that examine the distribution and connections of commissural interneurons. These experiments show differences in the rostrocaudal distribution of commissural interneurons [41] and differences in the synaptic organization of ipsi- and contralaterally projecting interneurons [42]. Ayali et al. experimentally showed differences in CPG output between blocking short ascending and descending connections, which further supports the idea of coupling asymmetry in the lamprey CPG [43]. From these results and our simulations, we hypothesize that intersegmental connections in the lamprey CPG exhibit nonuniform coupling asymmetry. This is an important insight into the CPG since individual intersegmental connection strengths are extremely difficult to measure experimentally.

Although the sinusoidal phase model agrees with the neural model for entrainment ranges as a function of the forcing position for both uniform and nonuniform coupling asymmetry, it does not capture all of the properties of entrainment ranges as a function of the forcing strength. In the sinusoidal phase model, entrainment ranges as a function of the forcing strength, $\alpha_{\mathrm{f}}$, are linear with slope depending on forcing position *m* and $\alpha_{k}/\alpha_{-k}$ [15]. As seen in Fig. 8, the derived phase model, even for stronger coupling, exhibits a nonlinear relationship between entrainment and forcing strength. This is especially evident along the lower bound of the entrainment range in Fig. 8(left). In addition to capturing the relationship between forcing strength and entrainment seen in the neural model, the derived phase model also captures the type of bifurcations that occur in the neural model. Namely, saddle-node bifurcations and Hopf bifurcations in the middle of the lower bound. The sinusoidal phase model only loses entrainment through saddle-node bifurcations [15]. Thus, our work justifies using a slightly more detailed phase model to approximate the neural model in further entrainment studies. We plan to further investigate entrainment of the lamprey CPG, both experimentally and computationally, by adding noisy perturbations to the deterministic bending signals.

In both the neural and the derived phase models, we chose parameter sets based on previous work [15, 29]. However, the entrainment results of both models approximately scale with the order of magnitude of coupling parameters. This is evident in Fig. 8. The two panels compare entrainment ranges as a function of the forcing position for two parameter sets which differ by a scale of 100. For the derived phase model, plotted in blue, the entrainment range on the right is exactly 100 times the entrainment range on the left. For the neural model, the entrainment ranges differ slightly in shape but the same change in magnitude is evident. This scaling also occurs in entrainment ranges as a function of the forcing position for both models. Thus, our results could be generalized to other models and parameter choices depending on the locomotion system being modeled.
